# Translating injury prevention evidence into safer padel: Protocol of a TRIPP-guided scoping review

**DOI:** 10.1371/journal.pone.0352442

**Published:** 2026-07-10

**Authors:** Lennert Goossens, Javier Ramos-Munell, Ana I. Fernandez-de-Osso, José Luis Ceballos-Sánchez

**Affiliations:** 1 Departamento de Educación y Deporte, Universidad Loyola Andalucía, Campus Sevilla, Dos Hermanas, Sevilla, España; 2 Departamento de Ciencias Sociales y de la Salud, Centro Universitario San Isidoro, Centro Adscrito a la Universidad Pablo de Olavide, Sevilla, España; Mayo Clinic’s Campus in Florida, UNITED STATES OF AMERICA

## Abstract

Padel is a rapidly growing sport with high injury incidence rates and substantial consequences. However, no comprehensive overview exists of the evidence on padel-related injuries and injury prevention strategies. A scoping review can help identify gaps in the emerging field of padel research. The objective of this scoping review is to systematically map the available evidence on padel‑related injuries and injury prevention strategies across all player populations and settings, from epidemiological description to intervention effectiveness. Studies describing injuries or injury risk factors or mechanisms in padel players of any kind and regarding primary injury prevention strategies or interventions involving stakeholders at any socio-ecological level will be considered. Studies that have been conducted in any geographic location, at any playing level, in any setting and in any implementation context will be considered. The proposed scoping review will be conducted in accordance with the JBI methodology for scoping reviews and the Preferred Reporting Items for Systematic Reviews and Meta-Analyses extension for Scoping Reviews (PRISMA-ScR) will be followed. A three-step search strategy will be utilized. The search strategy will be adapted for PubMed, SPORTDiscus, Web of Science, Scopus, EMBASE and CINAHL. A structured gray‑literature search will also be conducted. The data extracted from included papers will include specific details about the participants, concept, context, study methods and key findings relevant to the review questions. No critical appraisal of individual sources of evidence will be performed. The extracted data will be presented according to the six stages of the Translating Research into Injury Prevention Practice (TRIPP) framework. The review has been registered through Open Science Framework (osf.io/4t69j).

## Introduction

Padel is a relatively young racket sport played in doubles on an artificial turf court surrounded by walls. The sport’s high accessibility on a physical level, thanks to the relatively small court, makes padel popular among people of all ages and of various physical and motor ability levels. Moreover, it is one of the sports most played by women, who account for over 40% of all players. Recently, padel has experienced rapid growth worldwide. It is estimated that there were more than 35 million amateur padel players in 2025, and the professional padel circuit has grown from 6 international tournaments worldwide in 2019, to 318 in 2025. Consequently, padel courts are popping up everywhere across 150 countries [1]. Padel’s explosive global expansion has led to the ARISF status (Association of IOC-Recognized International Sports Federations) and has positioned it as a leading candidate for Olympic inclusion in Brisbane, 2032.

Evidence suggests that regular padel practice promotes health and is associated with improved physical fitness and body composition. However, since the sport is characterized by a high frequency of strokes and repetitive short-distance sprints in different directions, the physical demands of the sport are also substantial [2]. Unfortunately, this leads to a substantial injury burden, with incidence rates of 3 injuries per 1,000 training hours and 8 injuries per 1,000 matches, alongside prevalence estimates ranging from 40–95% across player levels. The elbow is the most frequently affected site, followed by the knee, shoulder, and lumbar regions, with tendon and muscle injuries comprising the majority [3]. Padel shows higher prevalence rates compared to tennis and squash, but a distinct profile favoring upper extremity overuse injuries [4]. Consequences of padel injuries include substantial time-loss (median 30 days) and risk of chronicity, underscoring the need for primary prevention [5,6].

Considering the adverse effects of injuries in padel players, primary prevention initiatives are necessary. Padel’s worldwide growth amplifies the urgency of evidence-based injury prevention strategies to support safe participation across recreational and elite levels. Research in other sports has proven that primary prevention strategies can significantly reduce the burden of sports injuries [7]. To develop prevention initiatives with a broader social impact, it is recommended to follow the phased approach of the Translating Research into Injury Prevention Programs (TRIPP) framework: document the injury epidemiology and etiology, develop the prevention program and study its efficacy, implementation context and effectiveness [8].

Currently, no comprehensive overview exists of the available evidence on padel-related injuries and injury prevention strategies. Consequently, padel lacks consensus guidelines or official primary prevention protocols from the International Padel Federation (FIP) or national federations, unlike other sports federations such as tennis’ ITF [9] or football’s FIFA, which has developed and validated a structured injury prevention program [10]. This gap leaves practitioners without evidence-based recommendations and researchers without clear direction for future studies. Padel research remains an emerging field with limited volume, heterogeneous study designs, and diverse outcomes that preclude formal meta-analysis or narrow efficacy questions at this stage. In line with contemporary JBI guidance [11], a scoping review was therefore selected as the most appropriate methodology for this review. Preliminary searches confirm a growing body of relevant studies that will meet our inclusion criteria.

A preliminary search of MEDLINE, the Cochrane Database of Systematic Reviews and JBI Evidence Synthesis was conducted on January 27, 2026, and no current or underway systematic reviews or scoping reviews on the topic were identified. One systematic review [3] and one scoping review [12] covering padel injuries have been published before, both with an exclusive focus on injury epidemiology. The absence of padel injury prevention reviews in Cochrane, JBI and MEDLINE suggests clear room for a new, TRIPP‑oriented scoping review including prevention strategies, efficacy and implementation aspects.

The main objective of this scoping review is to systematically map the available evidence on padel‑related injuries and injury prevention strategies across all player populations and settings, from epidemiological description to intervention effectiveness. Moreover, this review aims to identify research gaps and inform future studies. If applicable, evidence on effective prevention strategies will be used to inform real‑world implementation.

Following the PCC framework, the overarching question this scoping review seeks to answer is: ‘What is the extent, range and nature of research on injuries and injury prevention among padel players across all levels and settings?’. Aligned with the stages of the TRIPP framework, sub-questions include: (1) What is the incidence, prevalence, type, location and severity of injuries in padel players? (2) What are the risk factors, mechanisms and contexts that have been investigated in relation to padel injuries? (3) Which injury prevention strategies or interventions targeting padel players have been described, and what is known about their efficacy, feasibility and acceptability? (4) To what extent has the real‑world implementation of evidence-based padel injury programs been researched? These questions aim to map and describe the available evidence rather than to formally assess intervention effectiveness.

## Materials and methods

The proposed scoping review will be conducted in accordance with the JBI methodology for scoping reviews [11]. Moreover, the Preferred Reporting Items for Systematic Reviews and Meta-Analyses extension for Scoping Reviews (PRISMA-ScR) will be followed (S1 Table) [13]. The review has been registered through Open Science Framework (osf.io/4t69j).

### Inclusion criteria

In line with JBI guidance for scoping reviews, the PCC (Population, Concept, Context) framework will be used to define the eligibility criteria.

#### Participants.

For this review studies including padel players of any kind will be considered. No studies will be excluded based on the players’ age, gender or competition level. Moreover, studies regarding injury prevention strategies or interventions involving stakeholders at any socio-ecological level (e.g., coaches, parents, club owners, policy makers) will be considered.

#### Concept.

Studies describing injuries or injury risk factors or mechanisms in padel players will be considered. For this review, injury will be defined as tissue damage or other derangements of normal physical function due to participation in sports, resulting from rapid or repetitive transfer of kinetic energy [14]. Moreover, studies describing strategies or interventions aimed at the primary prevention of injuries in any population of padel players will be considered.

#### Context.

Studies that have been conducted in any geographic location, at any playing level (e.g., recreational, competition), in any setting (e.g., academy, public court, recreational club) and in any implementation context (e.g., club policies, coaching practices, preventive exercise programs, equipment recommendations) will be considered.

### Types of sources

For this scoping review both experimental and quasi-experimental study designs will be considered, including randomized controlled trials, non-randomized controlled trials, before and after studies and interrupted time-series studies. In addition, analytical observational studies including prospective and retrospective cohort studies, case-control studies and analytical cross-sectional studies will be considered for inclusion. Descriptive observational study designs including case series and descriptive cross-sectional studies will also be considered for inclusion. Qualitative studies that focus on data including barriers and facilitators for adoption or implementation of injury preventive measures will also be considered for inclusion. In addition, systematic reviews that meet the inclusion criteria may be used, depending on the research question, primarily as sources of primary studies. On the other hand, narrative and scoping reviews as well as non-peer-reviewed text and opinion papers will be excluded. Studies or reports where performance or biomechanics of padel was the sole focus, case reports, and newspaper and magazine articles will be excluded.

### Search strategy

The search strategy will aim to locate published studies as well as non–peer‑reviewed or not yet formally published studies (gray literature). A three-step search strategy will be utilized. First an initial limited search of PubMed and SPORTDiscus will be undertaken to identify articles on the topic. The eligibility criteria described above will be operationalized into a PICO (Population, Intervention, Comparator, Outcome) structure to guide the development of a sensitive and reproducible search strategy. The text words contained in the titles and abstracts of relevant articles, and the index terms used to describe the articles will be combined using appropriate Boolean logic and operators to develop a full search strategy for PubMed. The search strategy, including all identified keywords and index terms, will be adapted for each included database (PubMed, SPORTDiscus, Web of Science, Scopus, EMBASE, CINAHL Ultimate) (S2 Table). Lastly, the reference list of all articles selected for inclusion will be screened for additional studies. If necessary, authors of primary sources or reviews will be contacted for further information.

Studies published in any language and published from database inception to the present will be included.

In addition to database searches, a structured gray‑literature search will be conducted including theses/dissertations (ProQuest Dissertations & Theses Global; Tesis Doctorales en Red (TDR), TESEO, Dialnet tesis, Bielefeld Academic Search Engine (BASE), CORE, OpenDOAR), conference proceedings of major sport‑science and sports‑medicine societies (ECSS, ACSM, IOC World Conference on Prevention of Injury & Illness in Sport), websites of international and national padel federations and relevant sport‑governing bodies, clinical‑trial registries (ClinicalTrials.gov), and targeted Google searches.

### Study/source of evidence selection

Following the search, all identified citations will be collated and uploaded into Rayyan QCRI (Qatar Computing Research Institute), and duplicates will be removed. Following a pilot test, titles and abstracts will then be screened by four independent reviewers for assessment against the inclusion criteria for the review. Potentially relevant sources will be retrieved in full, and the full text of selected citations will be assessed in detail against the inclusion criteria by four independent reviewers. Reasons for exclusion of sources of evidence at full text review that do not meet the inclusion criteria will be recorded and reported in the scoping review. Any disagreements that arise between the reviewers at each stage of the selection process will be resolved through discussion, or with the involvement of an additional reviewer if necessary. The results of the search and the study inclusion process will be reported in full in the final scoping review and presented in a PRISMA-ScR flow diagram [15].

### Data extraction

Data will be extracted from papers included in the scoping review by four independent reviewers using a data extraction table developed by the reviewers based on a previous scoping review in the sports injury prevention domain [16]. The data extracted will include specific details about the participants, concept, context, study methods and key findings relevant to the review questions. A draft extraction table is provided (S3 Table). The draft data extraction table will be modified and revised as necessary during the process of extracting data from each included evidence source. Modifications will be detailed in the scoping review. Any disagreements that arise between the reviewers will be resolved through discussion, or with an additional reviewer. If appropriate, authors of papers will be contacted to request missing or additional data, where required.

No critical appraisal of individual sources of evidence will be performed. The proposed scoping review aims to map the extent, range and nature of available evidence on padel injuries and injury prevention, regardless of methodological quality, consistent with JBI guidance [11].

### Data analysis and presentation

The extracted data will be presented according to the six stages of the Translating Research into Injury Prevention Practice (TRIPP) framework. The TRIPP framework describes a six-step approach to guide scientific projects in the direction of effective injury prevention measures implementation. Through this approach, the available evidence will be systematically mapped, and descriptive and thematic analyses will be performed to identify existing knowledge and research gaps in padel injury epidemiology, risk factors, and prevention strategies. The assignment of each study to TRIPP stages will be done by consensus among reviewers and if applicable, a single study will be allowed to contribute to multiple stages. For articles of all TRIPP stages, study characteristics (design, number of participants, age, level of sport, geographical location, follow up/recall period, kind of publication), injury definition and registration, and data collection (if applicable) will be extracted. For TRIPP stage 1, injury frequencies and characteristics (severity, type, location) will be extracted. For TRIPP stage 2, injury risk factors and mechanisms will be extracted. For TRIPP stages 3 and 4, the preventive measure and delivery agent(s), as well as results, conclusions and recommendations will be extracted. For TRIPP stage 5, the preventive measure and delivery agent(s), and its implementation barriers, facilitators and behavioural determinants will be extracted. Finally, for TRIPP stage 6, the preventive measure and delivery agent(s), as well as results (Reach, Effectiveness, Adoption, Implementation, Maintenance), conclusions and recommendations will be extracted. Eventual graphical and/or tabular presentation of results will be accompanied by a narrative summary and will describe how the results relate to the review objectives and questions.

### Timeline

Record screening will be completed by May 31, 2026. Data extraction will be completed by June 30, 2026. Results are expected by October 31, 2026.

## Conclusion

This study presents the protocol for a scoping review that aims to systematically map the available evidence on padel‑related injuries and primary injury prevention. Given the incidence and consequences of injuries in padel, as well as the sport’s rapid global growth, primary prevention is a key area of focus. To date, however, no comprehensive overview has synthesized the existing evidence on padel injuries and preventive strategies. The proposed review will adhere to the JBI methodology for scoping reviews and the PRISMA‑ScR reporting guidelines. Findings will be organized according to the six stages of the TRIPP framework, enabling both descriptive and thematic analyses to identify current knowledge and research gaps in padel injury epidemiology, risk factors, and prevention approaches.

## Supporting information

S1 Table Preferred Reporting Items for Systematic reviews and Meta-Analyses extension for Scoping Reviews (PRISMA-ScR) Checklist. [1]. JBI = Joanna Briggs Institute; PRISMA-ScR = Preferred Reporting Items for Systematic reviews and Meta-Analyses extension for Scoping Reviews. * Where sources of evidence (see second footnote) are compiled from, such as bibliographic databases, social media platforms, and Web sites. † A more inclusive/heterogeneous term used to account for the different types of evidence or data sources (e.g., quantitative and/or qualitative research, expert opinion, and policy documents) that may be eligible in a scoping review as opposed to only studies. This is not to be confused with information sources (see first footnote). ‡ The frameworks by Arksey and O’Malley [2] and Levac and colleagues [3] and the JBI guidance [4,5] refer to the process of data extraction in a scoping review as data charting. § The process of systematically examining research evidence to assess its validity, results, and relevance before using it to inform a decision. This term is used for items 12 and 19 instead of “risk of bias” (which is more applicable to systematic reviews of interventions) to include and acknowledge the various sources of evidence that may be used in a scoping review (e.g., quantitative and/or qualitative research, expert opinion, and policy document).
(DOCX)

S2 Table Search strategy. (DOCX)

S3 Table Data extraction instrument. (DOCX)
