# Supplementary material for: Translating injury prevention evidence into safer padel: Protocol of a TRIPP-guided scoping review
Source: PLoS One. 2026 Jul 10;21(7):e0352442. doi: 10.1371/journal.pone.0352442 (PMC13353967; doi:10.1371/journal.pone.0352442)
Supplement: S2 Table — (DOCX) [file pone.0352442.s002.docx]

| PubMed | POPULATION   1. “Padel”.tw 2. “Paddle-tennis”.tw 3. “Padel-tennis”.tw 4. “padel tennis”.tw 5. “Padel player”.tw 6. “Padel game”.tw 7. “Padel match”.tw 8. “Padel categories”.tw 9. “Padel athletes”.tw 10. “Padel practitioners”.twm 11. “Professional padel”.tw 12. “Padel users”.tw 13. “Padel Racket”.tw   INTERVENTION  COMPARISON  OUTCOMES   1. Wounds and injuries/ 2. “Wounds and injuries”. tw 3. Injuries/ 4. “injur*”. tw 5. Wounds, Injury 6. Wound* 7. “Research-Related Injur*” 8. “Research Related Injur*” 9. Physical Trauma* 10. Trauma, Physical 11. Trauma 12. Abdominal injuries/ 13. “Abdominal injuries”.tw 14. “Hernia, Diaphragmatic, Traumatic”.tw 15. Arm injuries/ 16. “Arm injuries”.tw 17. “Upper body injuries”.tw 18. “Upper-body musculoskeletal injuries” 19. “elbow injuries”.tw 20. “Forearm injuries”.tw 21. “Humeral fractures”.tw 22. “Wrist injuries”.tw 23. Fracture Bone/ 24. "Ankle Fractures". tw 25. "Elbow Fractures". tw 26. "Femoral Fractures". tw 27. "Fibula Fractures". tw 28. "Fracture Dislocation". tw 29. "Fractures, Avulsion". tw 30. "Fractures, Closed". tw 31. "Fractures, Comminuted". tw 32. "Fractures, Compression". tw 33. "Fractures, Malunited". tw 34. "Fractures, Multiple". tw 35. "Fractures, Open". tw 36. "Fractures, Spontaneous". tw 37. "Fractures, Stress". tw 38. "Fractures, Ununited". tw 39. "Humeral Fractures". tw 40. "Intra-Articular Fractures". tw 41. "Knee Fractures". tw 42. "Osteoporotic Fractures". tw 43. "Periprosthetic Fractures". tw 44. "Radius Fractures". tw 45. "Rib Fractures". tw 46. "Shoulder Fractures". tw 47. "Skull Fractures". tw 48. "Spinal Fractures". tw 49. "Tibial Fractures". tw 50. "Ulna Fractures". tw 51. "Wrist Fractures". tw 52. Fractures, Cartilage/ 53. “Fractures, Cartilage”.tw 54. Hip injuries/ 55. “Hip injuries”.tw 56. “Hip dislocation”.tw 57. “lower body injuries”.tw 58. Leg injuries/ 59. “leg injuries”.tw 60. "Ankle Injuries". tw 61. "Foot Injuries". tw 62. "Knee Injuries". tw 63. "Tibial Meniscus Injuries". tw 64. Shoulder injuries/ 65. “Shoulder injuries”.tw 66. "Rotator Cuff Injuries". tw 67. "Shoulder Dislocation". tw 68. "Shoulder Impingement Syndrome". tw |
| --- | --- |
| EMBASE  FILTER  Limited to Article | POPULATION   1. 'padel'/exp 2. padel:ti,ab,kw 3. 'paddle tennis':ti,ab,kw 4. 'padel tennis':ti,ab,kw 5. 'padel-tennis':ti,ab,kw 6. 'padel player*':ti,ab,kw 7. 'padel game*':ti,ab,kw 8. 'padel match*':ti,ab,kw 9. 'padel categor*':ti,ab,kw 10. 'padel athlete*':ti,ab,kw 11. 'padel practitioner*':ti,ab,kw 12. 'professional padel':ti,ab,kw 13. 'padel user*':ti,ab,kw 14. 'padel racket*':ti,ab,kw   INTERVENTION  COMPARISON  OUTCOMES   1. 'injury'/exp 2. 'fracture'/exp 3. injur*:ti,ab,kw 4. wound*:ti,ab,kw 5. trauma*:ti,ab,kw 6. 'arm injur*':ti,ab,kw 7. 'upper body injur*':ti,ab,kw 8. 'elbow injur*':ti,ab,kw 9. 'humeral fracture*':ti,ab,kw 10. 'wrist injur*':ti,ab,kw 11. 'ankle fracture*':ti,ab,kw 12. 'elbow fracture*':ti,ab,kw 13. 'knee fracture*':ti,ab,kw 14. 'shoulder fracture*':ti,ab,kw 15. 'wrist fracture*':ti,ab,kw 16. 'hip injur*':ti,ab,kw 17. 'leg injur*':ti,ab,kw 18. 'ankle injur*':ti,ab,kw 19. 'knee injur*':ti,ab,kw 20. 'shoulder injur*':ti,ab,kw |
| SCOPUS  search: TITLE-ABS-KEY  Limited to Article | POPULATION   1. “Padel”.tw 2. “Paddle-tennis”.tw 3. “Padel-tennis”.tw 4. “padel tennis”.tw 5. “Padel player”.tw 6. “Padel game”.tw 7. “Padel match”.tw 8. “Padel categories”.tw 9. “Padel athletes”.tw 10. “Padel practitioners”.tw 11. “Professional padel”.tw 12. “Padel users”.tw 13. “Padel Racket”.tw   INTERVENTION  COMPARISON  OUTCOMES   1. “injur*”. tw 2. “Research-Related Injur*” 3. “Research Related Injur*” 4. Physical Trauma* 5. Trauma 6. “Abdominal injuries”.tw 7. “Arm injuries”.tw 8. “Upper body injuries”.tw 9. “Upper-body musculoskeletal injuries”. tw 10. “elbow injuries”.tw 11. “Forearm injuries”.tw 12. “Humeral fractures”.tw 13. “Wrist injuries”.tw 14. "Ankle Fractures". tw 15. "Elbow Fractures". tw 16. "Femoral Fractures". tw 17. "Fibula Fractures". tw 18. "Humeral Fractures". tw 19. "Intra-Articular Fractures". tw 20. "Knee Fractures". tw 21. "Osteoporotic Fractures". tw 22. "Periprosthetic Fractures". tw 23. "Radius Fractures". tw 24. "Rib Fractures". tw 25. "Shoulder Fractures". tw 26. "Skull Fractures". tw 27. "Spinal Fractures". tw 28. "Tibial Fractures". tw 29. "Ulna Fractures". tw 30. "Wrist Fractures". tw 31. “Fractures, Cartilage”.tw 32. “Hip injuries”.tw 33. “Hip dislocation”.tw 34. “lower body injuries”.tw 35. “leg injuries”.tw 36. "Ankle Injuries". tw 37. "Foot Injuries". tw 38. "Knee Injuries". tw 39. "Tibial Meniscus Injuries". tw 40. “Shoulder injuries”.tw 41. "Rotator Cuff Injuries". tw 42. "Shoulder Dislocation". tw 43. "Shoulder Impingement Syndrome". tw |
| WOS  search: TOPIC  Limited to Article | POPULATION   1. “Padel”.tw 2. “Paddle-tennis”.tw 3. “Padel-tennis”.tw 4. “padel tennis”.tw 5. “Padel player”.tw 6. “Padel game”.tw 7. “Padel match”.tw 8. “Padel categories”.tw 9. “Padel athletes”.tw 10. “Padel practitioners”.tw 11. “Professional padel”.tw 12. “Padel users”.tw 13. “Padel Racket”.tw   INTERVENTION  COMPARISON  OUTCOMES   1. “injur*”. tw 2. “Research-Related Injur*” 3. “Research Related Injur*” 4. Physical Trauma* 5. Trauma 6. “Abdominal injuries”.tw 7. “Arm injuries”.tw 8. “Upper body injuries”.tw 9. “Upper-body musculoskeletal injuries”. tw 10. “elbow injuries”.tw 11. “Forearm injuries”.tw 12. “Humeral fractures”.tw 13. “Wrist injuries”.tw 14. "Ankle Fractures". tw 15. "Elbow Fractures". tw 16. "Femoral Fractures". tw 17. "Fibula Fractures". tw 18. "Humeral Fractures". tw 19. "Intra-Articular Fractures". tw 20. "Knee Fractures". tw 21. "Osteoporotic Fractures". tw 22. "Periprosthetic Fractures". tw 23. "Radius Fractures". tw 24. "Rib Fractures". tw 25. "Shoulder Fractures". tw 26. "Skull Fractures". tw 27. "Spinal Fractures". tw 28. "Tibial Fractures". tw 29. "Ulna Fractures". tw 30. "Wrist Fractures". tw 31. “Fractures, Cartilage”.tw 32. “Hip injuries”.tw 33. “Hip dislocation”.tw 34. “lower body injuries”.tw 35. “leg injuries”.tw 36. "Ankle Injuries". tw 37. "Foot Injuries". tw 38. "Knee Injuries". tw 39. "Tibial Meniscus Injuries". tw 40. “Shoulder injuries”.tw 41. "Rotator Cuff Injuries". tw 42. "Shoulder Dislocation". tw 43. "Shoulder Impingement Syndrome". tw |
| CINAHL | POPULATION   1. "MH \"Racquet Sports Injuries\"" 2. "padel" 3. "paddle-tennis" 4. "padel-tennis" 5. "padel tennis" 6. "padel player" 7. "padel game" 8. "padel match" 9. "padel categories" 10. "padel athletes" 11. "padel practitioners" 12. "professional padel" 13. "padel users" 14. "padel racket"   INTERVENTION  COMPARISON  OUTCOMES   1. "MH \"Wounds and Injuries+\"" 2. "MH \"Fractures+\"" 3. "MH \"Tibial Fractures+\"" 4. "MH \"Elbow Fractures+\"" 5. "MH \"Shoulder Fractures+\"" 6. "MH \"Femoral Fractures+\"" 7. "MH \"Fractures, Ununited+\"" 8. "MH \"Fractures, Stress+\"" 9. "MH \"Radius Fractures+\"" 10. "MH \"Clavicle Fractures+\"" 11. "MH \"Wrist Fractures+\"" 12. "MH \"Knee Fractures+\"" 13. "MH \"Humeral Fractures+\"" 14. "MH \"Hand Fractures+\"" 15. "MH \"Hip Fractures+\"" 16. "wound*" 17. "injur*" 18. "trauma" 19. "physical trauma*" 20. "research-related injur*" 21. "research related injur*" 22. "abdominal injur*" 23. "traumatic diaphragmatic hernia" 24. "arm injur*" 25. "upper body injur*" 26. "upper-body musculoskeletal injur*" 27. "elbow injur*" 28. "forearm injur*" 29. "humeral fracture*" 30. "wrist injur*" 31. "fracture*" 32. "ankle fracture*" 33. "elbow fracture*" 34. "femoral fracture*" 35. "fibula fracture*" 36. "fracture dislocation*" 37. "avulsion fracture*" 38. "closed fracture*" 39. "comminuted fracture*" 40. "compression fracture*" 41. "malunited fracture*" 42. "multiple fracture*" 43. "open fracture*" 44. "spontaneous fracture*" 45. "stress fracture*" 46. "ununited fracture*" 47. "intra-articular fracture*" 48. "knee fracture*" 49. "osteoporotic fracture*" 50. "periprosthetic fracture*" 51. "radius fracture*" 52. "rib fracture*" 53. "shoulder fracture*" 54. "skull fracture*" 55. "spinal fracture*" 56. "tibial fracture*" 57. "ulna fracture*" 58. "wrist fracture*" 59. "cartilage fracture*" 60. "hip injur*" 61. "hip dislocation*" 62. "lower body injur*" 63. "leg injur*" 64. "ankle injur*" 65. "foot injur*" 66. "knee injur*" 67. "tibial meniscus injur*" 68. "shoulder injur*" 69. "rotator cuff injur*" 70. "shoulder dislocation" 71. "shoulder impingement syndrome" |
| SPORTIDISCUS | POPULATION   1. DE "PADDLE tennis" OR 2. DE "RACKETS (Sporting goods)" OR 3. DE "TENNIS rackets" OR 4. DE "RACKETS (Game)" OR 5. "padel"[Text Word] OR 6. "paddle-tennis"[Text Word] OR 7. "padel-tennis"[Text Word] OR 8. "padel tennis"[Text Word] OR 9. "padel player"[Text Word] OR 10. "padel game"[Text Word] OR 11. "padel match"[Text Word] OR 12. "padel categories"[Text Word] OR 13. "padel athletes"[Text Word] OR 14. "padel practitioners"[Text Word] OR 15. "professional padel"[Text Word] OR 16. "padel users"[Text Word] OR 17. "padel racket"[Text Word]   INTERVENTION  COMPARISON  OUTCOMES   1. DE "WOUNDS & injuries" OR 2. DE "BACKPACKING injuries" OR 3. DE "BLUNT trauma" OR 4. DE "BURNS & scalds" OR 5. DE "CHRONIC wounds & injuries" OR 6. DE "CRASH injuries" OR 7. DE "DECOMPRESSION sickness" OR 8. DE "DISABILITIES" OR 9. DE "HEAD injuries" OR 10. DE "JOINT dislocations" OR 11. DE "NERVOUS system injuries" OR 12. DE "ORGAN rupture" OR 13. DE "OVEREXERTION injuries" OR 14. DE "OVERUSE injuries" OR 15. DE "PENETRATING wounds" OR 16. DE "PHYSIOLOGIC strain" OR 17. DE "SOFT tissue injuries" OR 18. DE "SPORTS injuries" OR 19. DE "SUBLUXATION" OR 20. DE "WOUND care" OR 21. "injur*"[Text Word] OR 22. "Research Related Injur*"[Text Word] OR 23. "Abdominal injuries"[Text Word] OR 24. "Arm injuries"[Text Word] OR 25. "Upper body injuries"[Text Word] OR 26. "Upper-body musculoskeletal injuries"[Text Word] OR 27. "Elbow injuries"[Text Word] OR 28. "Forearm injuries"[Text Word] OR 29. "Humeral fractures"[Text Word] OR 30. "Wrist injuries"[Text Word] OR 31. "Ankle fractures"[Text Word] OR 32. "Elbow fractures"[Text Word] OR 33. "Femoral fractures"[Text Word] OR 34. "Fibula fractures"[Text Word] OR 35. "Fracture Dislocation"[Text Word] OR 36. "Intra-Articular Fractures"[Text Word] OR 37. "Knee Fractures"[Text Word] OR 38. "Osteoporotic Fractures"[Text Word] OR 39. "Periprosthetic Fractures"[Text Word] OR 40. "Radius fractures"[Text Word] OR 41. "Rib fractures"[Text Word] OR 42. "Shoulder fractures"[Text Word] OR 43. "Skull fractures"[Text Word] OR 44. "Spinal fractures"[Text Word] OR 45. "Tibial fractures"[Text Word] OR 46. "Ulna fractures"[Text Word] OR 47. "Wrist fractures"[Text Word] OR 48. "Fractures cartilage"[Text Word] OR 49. "Hip injuries"[Text Word] OR 50. "Hip dislocation"[Text Word] OR 51. "Lower body injuries"[Text Word] OR 52. "Leg injuries"[Text Word] OR 53. "Ankle injuries"[Text Word] OR 54. "Foot injuries"[Text Word] OR 55. "Knee injuries"[Text Word] OR 56. "Tibial meniscus injuries"[Text Word] OR 57. "Shoulder injuries"[Text Word] OR 58. "Rotator cuff injuries"[Text Word] OR 59. "Shoulder dislocation"[Text Word] OR 60. "Shoulder impingement syndrome"[Text Word] |

**Pubmed**

((((((((((((("padel"[Text Word]) OR ("paddle-tennis"[Text Word])) OR ("padel-tennis"[Text Word])) OR ("padel tennis"[Text Word])) OR ("padel player"[Text Word])) OR ("padel game"[Text Word])) OR ("padel match"[Text Word])) OR ("padel categories"[Text Word])) OR ("padel athletes"[Text Word])) OR ("padel practitioners"[Text Word])) OR ("professional padel"[Text Word])) OR ("padel users"[Text Word])) OR ("padel racket"[Text Word])) AND (((((((((((((((((((((((((((((((((((((((((((((((((((((((((((((Wounds and injuries[MeSH Terms]) OR (Injuries[MeSH Terms])) OR (Abdominal Injuries[MeSH Terms])) OR (Arm Injuries[MeSH Terms])) OR (Fracture Bone[MeSH Terms])) OR (Fractures, Cartilage[MeSH Terms])) OR (Hip injuries[MeSH Terms])) OR (Leg injuries[MeSH Terms])) OR (Shoulder Injuries[MeSH Terms])) OR ("wounds and injuries"[Text Word])) OR ("injur*"[Text Word])) OR ("wounds,injury"[Text Word])) OR ("research-related injur*"[Text Word])) OR ("research related injur*"[Text Word])) OR ("physical trauma*"[Text Word])) OR ("trauma, physical"[Text Word])) OR ("trauma"[Text Word])) OR ("abdominal injuries"[Text Word])) OR ("hernia, diaphragmatic, traumatic"[Text Word])) OR ("arm injuries"[Text Word])) OR ("upper body injuries"[Text Word])) OR ("upper-body musculoskeletal injuries"[Text Word])) OR ("elbow injuries"[Text Word])) OR ("forearm injuries"[Text Word])) OR ("humeral fractures"[Text Word])) OR ("wrist injuries"[Text Word])) OR ("fracture bone"[Text Word])) OR ("ankle fractures"[Text Word])) OR ("elbow fractures"[Text Word])) OR ("femoral fractures"[Text Word])) OR ("fibula fractures"[Text Word])) OR ("fracture dislocation"[Text Word])) OR ("fractures, avulsion"[Text Word])) OR ("fractures, closed"[Text Word])) OR ("fractures, comminuted"[Text Word])) OR ("fractures compression"[Text Word])) OR ("fractures, malunited"[Text Word])) OR ("fractures, stress"[Text Word])) OR ("humeral fractures"[Text Word])) OR ("knee fractures"[Text Word])) OR ("radius fractures"[Text Word])) OR ("rib fractures"[Text Word])) OR ("shoulder fractures"[Text Word])) OR ("skull fractures"[Text Word])) OR ("spinal fractures"[Text Word])) OR ("tibial fractures"[Text Word])) OR ("ulna fractures"[Text Word])) OR ("wrist fractures"[Text Word])) OR ("fractures, cartilage"[Text Word])) OR ("hip injuries"[Text Word])) OR ("hip dislocation"[Text Word])) OR ("lower body injuries"[Text Word])) OR ("leg injuries"[Text Word])) OR ("ankle injuries"[Text Word])) OR ("foot injuries"[Text Word])) OR ("knee injuries"[Text Word])) OR ("tibial meniscus injuries"[Text Word])) OR ("shoulder injuries"[Text Word])) OR ("rotator cuff injuries"[Text Word])) OR ("shoulder dislocation"[Text Word])) OR ("shoulder impingement syndrome"[Text Word]))

**SCOPUS**

(TITLE-ABS-KEY("padel" OR "paddle-tennis" OR "padel-tennis" OR "padel tennis" OR "padel player" OR "padel players" OR "padel athletes" OR "padel practitioners") AND TITLE-ABS-KEY(injur* OR wound* OR trauma* OR "physical trauma" OR fracture* OR dislocation* OR "musculoskeletal injur*" OR "overuse injur*" OR "acute injur*" OR "sport injur*" OR "shoulder injur*" OR "elbow injur*" OR "wrist injur*" OR "hip injur*" OR "knee injur*" OR "ankle injur*" OR "foot injur*" OR "leg injur*" OR "arm injur*" OR "upper limb injur*" OR "lower limb injur*" OR "rotator cuff"))

**EMBASE**

(('padel/exp OR ''paddle tennis' OR 'padel tennis' OR 'padel-tennis':OR 'padel player*' OR 'padel game*' OR 'padel match*' OR 'padel categor*' OR 'padel athlete*' OR 'padel practitioner*' OR 'professional padel' OR 'padel user*' OR 'padel racket*') AND ('injury'/exp OR 'fracture'/exp OR 'injur*' OR 'wound*' OR 'trauma*' OR 'arm injur*' OR 'upper body injur*' OR 'elbow injur*'OR 'humeral fracture*' OR 'wrist injur*' OR 'ankle fracture*' OR 'elbow fracture*' OR 'knee fracture*' OR 'shoulder fracture*' OR 'wrist fracture*' OR 'hip injur*' OR 'leg injur*' OR 'ankle injur*' OR 'knee injur*' OR 'shoulder injur*'))

**WOS**

Query 1:

TS=("padel" OR "paddle-tennis" OR "padel-tennis" OR "padel tennis" OR "padel player" OR "padel game" OR "padel match" OR "padel categories" OR "padel athletes" OR "padel practitioners")

Query 2:

TS=(("Injuries" OR "Research Related Injur*" OR "Abdominal injuries" OR "Arm injuries" OR "Upper body injuries" OR "Upper-body musculoskeletal injuries" OR "Elbow injuries" OR "Forearm injuries" OR "Humeral fractures" OR "Wrist injuries" OR "Ankle fractures" OR "Elbow fractures" OR "Femoral fractures" OR "Fibula fractures" OR "Fracture Dislocation" OR "Intra-Articular Fractures" OR "Knee Fractures" OR "Osteoporotic Fractures" OR "Periprosthetic Fractures" OR "Radius fractures" OR "Rib fractures" OR "Shoulder fractures" OR "Skull fractures" OR "Spinal fractures" OR "Tibial fractures" OR "Ulna fractures" OR "Wrist fractures" OR "Fractures cartilage" OR "Hip injuries" OR "Hip dislocation" OR "Lower body injuries" OR "Leg injuries" OR "Ankle injuries" OR "Foot injuries" OR "Knee injuries" OR "Tibial meniscus injuries" OR "Shoulder injuries" OR "Rotator cuff injuries" OR "Shoulder dislocation" OR "Shoulder impingement syndrome" ))

**CINAHL**

(MH "Racquet Sports Injuries" OR "padel" OR "paddle-tennis" OR "paddle-tennis" OR "padel tenis" OR "padel player" OR "padel game" OR "padel match" OR "padel categories" OR "padel athletes" OR "padel practioners" OR "proffesional padel" OR "padel users" OR "padel racket") AND MH "Wounds and Injuries+" OR MH "Fractures+" OR MH "Tibial Fractures+" OR MH "Elbow Fractures+" OR MH "Shoulder Fractures+" OR MH "Femoral Fractures+" OR MH "Fractures, Ununited+" OR MH "Fractures, Stress+" OR MH "Radius Fractures+" OR MH "Clavicle Fractures+" OR MH "Wrist Fractures+" OR MH "Knee Fractures+" OR MH "Humeral Fractures+" OR MH "Hand Fractures+" OR MH "Hip Fractures+" "wound*" OR "injur*"  OR "trauma"  OR "physical trauma*"  OR "research-related injur*"  OR "research related injur*" OR "abdominal injur*" OR "traumatic diaphragmatic hernia" OR "arm injur*" OR "upper body injur*" OR "upper-body musculoskeletal injur*" OR "elbow injur*" OR "forearm injur*" OR "humeral fracture*" OR "wrist injur*" OR fracture* OR "ankle fracture*" OR "elbow fracture*" OR "femoral fracture*" OR "fibula fracture*" OR "fracture dislocation*"OR "avulsion fracture*"OR "closed fracture*"OR "comminuted fracture*"OR "compression fracture*"OR "malunited fracture*"OR "multiple fracture*"OR "open fracture*"OR "spontaneous fracture*"OR "stress fracture*"OR "ununited fracture*"OR "intra-articular fracture*"OR "knee fracture*"OR "osteoporotic fracture*"OR "periprosthetic fracture*"OR "radius fracture*"OR "rib fracture*"OR "shoulder fracture*"OR "skull fracture*"OR "spinal fracture*"OR "tibial fracture*"OR "ulna fracture*"OR "wrist fracture*"OR "cartilage fracture*"OR "hip injur*"OR "hip dislocation*"OR "lower body injur*"OR "leg injur*"OR "ankle injur*"OR "foot injur*" OR "knee injur*" OR "tibial meniscus injur*" OR "shoulder injur*" OR "rotator cuff injur*" OR "shoulder dislocation*" OR "shoulder impingement syndrome"

**SPORTDISCUS**

DE "PADDLE tennis" OR DE "RACKETS (Sporting goods)" OR DE "TENNIS rackets" OR DE "RACKETS (Game)" OR "padel"[Text Word] OR"paddle-tennis"[Text Word] OR"padel-tennis"[Text Word] OR"padel tennis"[Text Word] OR"padel player"[Text Word] OR"padel game"[Text Word] OR"padel match"[Text Word] OR"padel categories"[Text Word] OR"padel athletes"[Text Word] OR"padel practitioners"[Text Word] OR"professional padel"[Text Word] OR"padel users"[Text Word] OR"padel racket"[Text Word] AND DE "WOUNDS & injuries" OR DE "BACKPACKING injuries" OR DE "BLUNT trauma" OR DE "BURNS & scalds" OR DE "CHRONIC wounds & injuries" OR DE "CRASH injuries" OR DE "DECOMPRESSION sickness" OR DE "DISABILITIES" OR DE "HEAD injuries" OR DE "JOINT dislocations" OR DE "NERVOUS system injuries" OR DE "ORGAN rupture" OR DE "OVEREXERTION injuries" OR DE "OVERUSE injuries" OR DE "PENETRATING wounds" OR DE "PHYSIOLOGIC strain" OR DE "SOFT tissue injuries" OR DE "SPORTS injuries" OR DE "SUBLUXATION" OR DE "WOUND care" OR "injur*"[Text Word] OR"Research Related Injur*"[Text Word] OR"Abdominal injuries"[Text Word] OR"Arm injuries"[Text Word] OR"Upper body injuries"[Text Word] OR"Upper-body musculoskeletal injuries"[Text Word] OR"Elbow injuries"[Text Word] OR"Forearm injuries"[Text Word] OR"Humeral fractures"[Text Word] OR"Wrist injuries"[Text Word] OR"Ankle fractures"[Text Word] OR"Elbow fractures"[Text Word] OR"Femoral fractures"[Text Word] OR"Fibula fractures"[Text Word] OR"Fracture Dislocation"[Text Word] OR"Intra-Articular Fractures"[Text Word] OR"Knee Fractures"[Text Word] OR"Osteoporotic Fractures"[Text Word] OR"Periprosthetic Fractures"[Text Word] OR"Radius fractures"[Text Word] OR"Rib fractures"[Text Word] OR"Shoulder fractures"[Text Word] OR"Skull fractures"[Text Word] OR"Spinal fractures"[Text Word] OR"Tibial fractures"[Text Word] OR"Ulna fractures"[Text Word] OR"Wrist fractures"[Text Word] OR"Fractures cartilage"[Text Word] OR"Hip injuries"[Text Word] OR"Hip dislocation"[Text Word] OR"Lower body injuries"[Text Word] OR"Leg injuries"[Text Word] OR"Ankle injuries"[Text Word] OR"Foot injuries"[Text Word] OR"Knee injuries"[Text Word] OR"Tibial meniscus injuries"[Text Word] OR"Shoulder injuries"[Text Word] OR"Rotator cuff injuries"[Text Word] OR"Shoulder dislocation"[Text Word] OR"Shoulder impingement syndrome"[Text Word]
