# Supplementary material for: Translating injury prevention evidence into safer padel: Protocol of a TRIPP-guided scoping review
Source: PLoS One. 2026 Jul 10;21(7):e0352442. doi: 10.1371/journal.pone.0352442 (PMC13353967; doi:10.1371/journal.pone.0352442)
Supplement: S3 Table — (DOCX) [file pone.0352442.s003.docx]

TRIPP stage 1

| Author | Study information | Injury definition and data collection | Injury frequencies and characteristics |
| --- | --- | --- | --- |
| Author(s) (year) | Design:  Number:  Age:  Level of sport:  Location:  Follow up/Recall:  Kind of publication: | Injury definition:  Injury registration:  Data collection: | Injury frequencies:  Injury severity:  Injury type:  Injury location: |

TRIPP stage 2

| Author | Study information | Injury definition and data collection | Risk factors and mechanisms |
| --- | --- | --- | --- |
| Author(s) (year) | Design:  Number:  Age:  Level of sport:  Location:  Follow up/Recall:  Kind of publication: | Injury definition:  Injury registration:  Data collection: | Risk factors:  Mechanisms: |

TRIPP stage 3-4

| Author | Study information | Injury definition and data collection | Preventive measure | Results/ Conclusion/ Recommendations |
| --- | --- | --- | --- | --- |
| Author(s) (year) | Design:  Number:  Age:  Level of sport:  Location:  Follow up/Recall:  Kind of publication: | Injury definition:  Injury registration:  Data collection: | Preventive measure:  Delivery agent(s): | Results:  Conclusion:  Recommendations: |

TRIPP stage 5

| Author | Study information | Injury definition | Preventive measure | Intervention context |
| --- | --- | --- | --- | --- |
| Author(s) (year) | Design:  Number:  Age:  Level of sport:  Location:  Follow up/Recall:  Kind of publication: | Injury definition: | Preventive measure:  Delivery agent(s): | Barriers:  Facilitators:  Behavioural determinants: |

TRIPP stage 6

| Author | Study information | Injury definition and data collection | Preventive measure | Results/ Conclusion/ Recommendations |
| --- | --- | --- | --- | --- |
| Author(s) (year) | Design:  Number:  Age:  Level of sport:  Location:  Follow up/Recall:  Kind of publication: | Injury definition:  Injury registration:  Data collection: | Preventive measure:  Delivery agent(s): | Results (Reach, Effectiveness, Adoption, Implementation, Maintenance):  Conclusion:  Recommendations: |
